# Acheron/Larp6 Is a Survival Protein That Protects Skeletal Muscle From Programmed Cell Death During Development

**DOI:** 10.3389/fcell.2020.00622

**Published:** 2020-07-29

**Authors:** Ankur Sheel, Rong Shao, Christine Brown, Joanne Johnson, Alexandra Hamilton, Danhui Sun, Julia Oppenheimer, Wendy Smith, Pablo E. Visconti, Michele Markstein, Carol Bigelow, Lawrence M. Schwartz

**Affiliations:** ^1^Department of Biology, University of Massachusetts Amherst, Amherst, MA, United States; ^2^Molecular and Cellular Biology Program, University of Massachusetts Amherst, Amherst, MA, United States; ^3^Department of Pharmacology, School of Medicine, Shanghai Jiao Tong University, Shanghai, China; ^4^Department of Biology, Barnard College, Columbia University, New York, NY, United States; ^5^Department of Biology, College of Science, Northeastern University, Boston, MA, United States; ^6^Department of Veterinary and Animal Sciences, University of Massachusetts Amherst, Amherst, MA, United States; ^7^Department of Biostatistics and Epidemiology, School of Public Health and Health Sciences, University of Massachusetts Amherst, Amherst, MA, United States

**Keywords:** *Manduca sexta*, intersegmental muscle, *Drosophila*, BAD, apoptosis, autophagy, cytochrome *c*, BBH1

## Abstract

The term programmed cell death (PCD) was coined in 1965 to describe the loss of the intersegmental muscles (ISMs) of moths at the end of metamorphosis. While it was subsequently demonstrated that this hormonally controlled death requires *de novo* gene expression, the signal transduction pathway that couples hormone action to cell death is largely unknown. Using the ISMs from the tobacco hawkmoth *Manduca sexta*, we have found that *Acheron/LARP6* mRNA is induced ∼1,000-fold on the day the muscles become committed to die. Acheron functions as a survival protein that protects cells until cell death is initiated at eclosion (emergence), at which point it becomes phosphorylated and degraded in response to the peptide Eclosion Hormone (EH). Acheron binds to a novel BH3-only protein that we have named BBH1 (BAD/BNIP3 homology 1). BBH1 accumulates on the day the ISMs become committed to die and is presumably liberated when Acheron is degraded. This is correlated with the release and rapid degradation of cytochrome *c* and the subsequent demise of the cell. RNAi experiments in the fruit fly *Drosophila* confirmed that loss of Acheron results in precocious ecdysial muscle death while targeting BBH1 prevents death altogether. Acheron is highly expressed in neurons and muscles in humans and drives metastatic processes in some cancers, suggesting that it may represent a novel survival protein that protects terminally differentiated cells and some cancers from death.

## Introduction

Virtually all cells express the genetic machinery required to commit suicide, a process known as programmed cell death (PCD). The ability to selectively target individual cells empowers the organism with a great deal of developmental plasticity. Cell death can be used to match populations of interacting cells, such as motor neurons and the skeletal muscles they innervate (Hamburger, 1975). It can sculpt portions of the body, for example when parallel rows of cells die in the developing limb bud to free up the digits (Lorda-Diez et al., 2015). Cell death can also remove deleterious cells, such as self-reactive thymocytes during negative selection in the thymus (McPhee et al., 1979). Lastly, it can also recycle obsolete tissues such as the tadpole tail during amphibian metamorphosis (Ishizuya-Oka, 2011). Unfortunately, defects in the regulation of PCD can have devastating consequences for health when either valuable cells are lost (e.g., neurodegenerative disorders) or deleterious ones are allowed to persist (e.g., autoimmunity and cancer). In fact, it has been estimated that misregulation of cell death may contribute to 70% of human diseases (Reed, 2002).

The best characterized cell death program, apoptosis, involves cellular shrinkage, membrane blebbing, chromatin condensation, and the degradation of genomic DNA into oligonucleosomal fragments that form the classic “DNA ladder” on agarose gels (Kerr et al., 1972; Wyllie, 1980; Miura et al., 1993). Other cells, most notably terminally differentiated cells like muscles and neurons, often undergo a distinct process that has been referred to as type II degeneration or autophagic cell death (Chu-Wang and Oppenheim, 1978; Schwartz et al., 1993; Denton and Kumar, 2019). Other than shrinking, these cells do not display the classic behavior of apoptosis. Instead, their most notable feature is a dramatic increase in the number of autophagic vesicles that facilitate the degradation of bulk cytoplasm and whole organelles such as mitochondria (Ashford and Porter, 1962; Youle and Narendra, 2011). It has been suggested that the term “cell death with autophagy” or “autophagy-dependent cell death” might be more appropriate descriptors given that many cells can undergo autophagy without dying (Kroemer and Levine, 2008; Shen et al., 2012; Galluzzi et al., 2018). At present, the molecular mechanisms that mediate autophagy dependent cell death are largely unknown.

The term PCD was coined by Lockshin and Williams in 1965 to describe the loss of the intersegmental muscles (ISMs) of moths at the end of metamorphosis (Lockshin and Williams, 1965). These giant muscles are used by the developing adult moth to eclose (emerge) at adult eclosion and then undergo an autophagic death during the subsequent 30 h (Kuwana, 1936; Lockshin and Williams, 1965; Beaulaton and Lockshin, 1977). The timing of ISM death is carefully orchestrated by the endocrine system. The ISMs acquire the competence to die when the circulating levels of the insect molting hormone 20-hydroxyecdysone (20E) decline below a specific threshold late on day 17 of pupal–adult development (the day before adult eclosion) (Truman and Riddiford, 1970; Schwartz and Truman, 1983). A further decline in 20E then determines the timing of Eclosion Hormone (EH) release late on day 18, which then triggers both the eclosion behavior and ISM death about a half hour before sunset (lights out) [0 h post-eclosion (PE)] on day 18 of pupal–adult development. EH acts on the ISMs to dramatically induce the production of the second messenger cyclic guanosine monophosphate (cGMP), an essential step in triggering ISM death (Schwartz and Truman, 1984). However, the subsequent signal transduction events that mediate ISM death are largely unknown except that the process is dependent on *de novo* gene expression earlier on day 18 (Schwartz et al., 1990). Treating animals on the day preceding adult eclosion with inhibitors of RNA or protein synthesis blocks the subsequent demise of the ISMs. A number of genes are induced when the ISMs become committed to die, but the one(s) that are required for actually mediating death are unknown. Here we describe a new signal transduction cascade that couples EH action to the subsequent release and degradation of cytochrome *c*. The key player in this pathway is Acheron/LARP6, a novel survival protein that protects the cells until the moment that death is triggered, at which point it becomes phosphorylated and degraded. Given that Acheron regulates the differentiation and survival of neurons, muscles, and some cancers, this pathway may contribute to human development and pathogenesis (Wang et al., 2009; Shao et al., 2012).

## Materials and Methods

### Animals

The tobacco hawkmoth, *Manduca sexta*, was reared and staged as described previously (Schwartz and Truman, 1983). Abdomens were isolated from staged animals and pinned at length under ice cold saline. In some experiments, the ISMs were fixed overnight at 4°C with 4% paraformaldehyde for immunohistochemistry (described below). For biochemical studies, the ISMs were dissected free from all adhering tissue, flash frozen on dry ice, and stored in liquid nitrogen until needed.

### RNA-Seq and RNA Quantification

The ISMs of three to four animals per developmental stage (eight time points in total: days 13, 14, 15, 16, 17, 18, and 1-h PE; plus 20-hydroxyecdysone injection on day 17: 20E) were homogenized and total RNA was isolated with the MirVana kit (Ambion) according to manufacturer’s instructions. cDNA libraries were constructed with total RNA, analyzed with a Bioanalyzer (Agilent Technologies; Santa Clara, CA, United States) and 50SE RNA-Seq was performed on an Illumina HiSeq^TM^ 2000 (San Diego, CA, United States) by Beijing Genomics Institute (Hong Kong) (Tsuji et al., 2020). Gene and protein sequences were analyzed using Manduca Base^1^ and the revised-OGS-June 2012 dataset. All the sequencing libraries are accessible from Gene Expression Omnibus (GEO) (accession number GSE80830).

In separate experiments, cDNA was synthesized with SuperScript III reverse transcriptase (Invitrogen) and qPCR performed on a Stratagene Mx3000P machine (Agilent Technologies) using SYBR Select Master Mix (Applied Biosystems). Samples were run in duplicate or triplicate and the constitutively expressed ubiquitin-fusion 80 (Ubf80) mRNA was used as a reference gene for normalization. Relative change in gene expression was determined using the ΔΔCt method. Primer sequences are in the table below.

### Western Blotting

Proteins from staged ISMs were extracted in Laemmli buffer and 20 μg of each sample was fractionated using either 4–15% gradient (Bio-Rad) or 10% polyacrylamide gel electrophoresis. Proteins were transferred to Immobilon-P (EMD Millipore) and then incubated with rabbit polyclonal antisera generated against: *Manduca* Acheron (1:1000; below); cytochrome c (1:1000; BD Pharmingen #556433); BAD (1:2000; LifeSpan BioSciences #LS-C63129); or tubulin (E7) (1:2000; Developmental Studies Hybridoma Bank). The anti-*Manduca* Acheron antibody was produced in rabbits. A portion of Acheron cDNA encoding amino acids 18–409 was cloned into the pBluescript KS vector (Agilent Technologies) and then subcloned into the pMALTM vector (New England Biolabs) to create a fusion protein with maltose binding protein. Protein expression was induced with isopropyl β-D-1-thiogalactopyranoside (IPTG) and the crude extract was purified by passage over an amylose resin column and elution with maltose. The maltose binding protein was then removed from the Acheron protein by Factor Xa cleavage (New England Biolabs). Approximately 200 μg protein in Freund’s adjuvant was injected into rabbits (Pocono Rabbit Farm), followed by boosting and serum collection over several weeks. The crude serum was purified over desalting and IgG purification columns (BioRad) prior to use on Western blots. An HRP-labeled goat anti-rabbit (Jackson ImmunoResearch) and an ECL kit (Pierce) were used to detect the primary antibody binding.

To evaluate the binding of the rabbit anti-mouse BAD antibody (LifeSpan BioSciences # LS-C63129) to BBH1 from *Manduca*, we subcloned amino acids 83–115 into the pET100/D-TOPO vector (ThermoFisher) and transfected it into BL21 *Escherichia coli*. Expression was induced by treating the cells with IPTG.

### Immunohistochemistry

Paraformaldehyde-fixed ISMs were embedded in paraffin and cut lengthwise to 4 μm sections. Both pre- and PE ISM sections were affixed to the same glass slides to insure identical processing. Sections were deparaffinized and rehydrated and antigen retrieval was performed with a sodium citrate buffer (10 mM; plus 0.05% Tween-20, pH 6.0) for 10 min. After 1 h incubation in blocking solution (1% BSA, 0.05%Triton-x, 10% normal goat serum in PBS), sections were incubated in primary antibodies (anti-cytochrome *c*, 1:100, BD Pharmingen #556433; anti-Acheron, 1:100) overnight at 4°C. Sections were then treated with 0.3% H_2_O_2_ to block endogenous peroxidase. A biotinylated secondary antibody (1:100, Vector Laboratories) was applied, followed by incubation in ABC reagent and diaminobenzidine (DAB) chromogen (Vector Laboratories) for signal visualization.

### *In vitro* Acheron Phosphorylation and Degradation Assays

Frozen ISMs were homogenized in lysis buffer: 10 mM Tris pH 7.4, 1% Triton X-100, 0.5% NP-40, 150 mM NaCl, 20 mM NaF, 0.2 mM Na_3_VO_4_, 1 mM EDTA, 1 mM EGTA, and 0.2 mM PMSF. Lysates from day 18 muscles (100 μg) or 1 h PE (200 μg) were incubated with kinase phosphorylation buffer containing: 30 mM MgCl_2_, 1.5 mM DTT, 75 mM beta-glycerophosphate, 0.15 mM Na_3_VO_4_, 3.75 mM EGTA, and 0.3 mM ATP in the presence or absence of 100 μM cGMP, 10 μM 1-isobutyl-3-methylxanthine (IBMX), and 1× STOP phosphatase inhibitors (Roche) for 1 h at room temperature. Samples were then boiled in Laemmli buffer, fractionated by 10% SDS-PAGE, and then transferred onto a PVDF membrane and reacted with an anti-Acheron antibody. For phosphorylation assays, the same methods were used except that: (1) 1 mCi γ^32^P-ATP (Perkin Elmer) replaced the unlabeled ATP and (2) the PVDF membrane was used for film autoradiography prior to processing for Western blotting.

### Co-immunoprecipitation Assays

Assays employed either flash frozen day 18 ISM extracts or mouse C_2_C_12_ myoblast lysates. C_2_C_12_ cells (American Type Culture Collection) were cultured in Dulbecco’s modified Eagle medium (DMEM) supplemented with 15% (V/V) calf serum (CS), 5% fetal bovine serum (FBS, Atlanta Biologicals), and 100 units/ml of penicillin-streptomycin (Gibco). Cell lysates (400–500 μg) were mixed at 4°C overnight with a rabbit polyclonal antibody (1:200) against Acheron (Wang et al., 2009) in 500 μl of immunoprecipitation buffer containing:10 mM Tris pH 7.4, 1% triton, 0.5% NP-40, 150 mM NaCl, 20 mM NaF, 0.2 mM Na_3_VO_4_, 1 mM EDTA, 1 mM EGTA, and 0.2 mM PMSF. The immunocomplex was incubated with protein A sepharose CL-4B (GE Healthcare Life Sciences) at 4°C for 4 h followed by extensive washing. The samples were then subjected to immunoblotting using Bcl2 (1:100, Santa Cruz #sc-7382), BAD (1: 200, # LS-C63129, LifeSpan BioSciences), CASK (1:400, Santa Cruz #sc-13158), or Bax (1:100, Santa Cruz #sc-7480) antibodies. Each co-IP assay was performed two to four times.

### C_2_C_12_ Cell Hypoxia

C_2_C_12_ cells were cultured as described above and then incubated with or without 100 μM CoCl_2_ for 24 h. Cells were extracted in RIPA buffer (50 mM Tris pH 8.0, 0.5% NP-40, 150 mM NaCl, 5 mM EDTA, 0.5% sodium deoxycholate, 0.1% SDS) plus Roche cOmplete Protease Inhibitor Cocktail and then subjected to Western blotting as described above. Anti-BNIP3 (ELabScience #ESAP11106) was used at 1:2000.

### Protein Sequence Analyses

The *Manduca* BBH1 protein sequence GEO (GEO number: MT777178) was computationally compared to mouse BAD (Bcl2-Associated Agonist of Cell Death isoform 2; NCBI Reference Sequence: NP_001272382.1) and BNIP3 (Adenovirus E1B 19 kDa Protein-Interacting Protein 3; NCBI Reference Sequence: NP_033890.1) using the Clustal Omega alignment tool with the default values (Sievers et al., 2011). Conserved amino acid substitutions were noted manually using the Blosum62 substitution matrix (Henikoff and Henikoff, 1992).

### *Drosophila* Experiments

Transgenic flies expressing DMEF (*Drosophila* Myocyte Enhancer Factor 2) driven red fluorescent protein (RFP) with or without *Drosophila* Acheron RNAi [CG17386/also known as Achilles in *Drosophila* (Li et al., 2017) (or BBH1 (CG5059)] under the control of the Gal4 upstream activating sequence (UAS) were crossed to the DMEF-Gal4 driver line, which is expressed in all muscles. Flies were maintained at 25°C with a 12 h light/dark cycle, and both males and females were used in each experiment. Flies were shifted to 29°C as pupae and the pharate adults examined via fluorescence microscopy. The percentage of flies that eclosed successfully was documented for each genotype. The loss of the ptilinal muscles in the head was evaluated by examining muscle fluorescence with a dissecting microscope. Flies were obtained from the Bloomington *Drosophila* Stock Center.

### Statistical Analysis

For the RNA-seq data analysis, we assessed the statistical significance for changes in gene expression across development using the Kruskal–Wallis test and summarized the results with scatter plots of the raw data, together with overlays of means. Group differences in the trajectories of reads per kilobase million (RPKM) over time were assessed through the fitting of piecewise linear regression models, as appropriate to avoid overfitting. For analysis of the qPCR data, we employed the one-tailed Student’s *t*-test and considered a *p*-value < 0.05 to be statistically significant. Group differences in percent of *Drosophila* that eclosed were assessed for statistical significance using the two sample test of equality of binomial proportions. All analyses were performed in Stata *version 14* (StataCorp, 2015).

## Results

### Acheron Expression During Development

**FIGURE 1:**
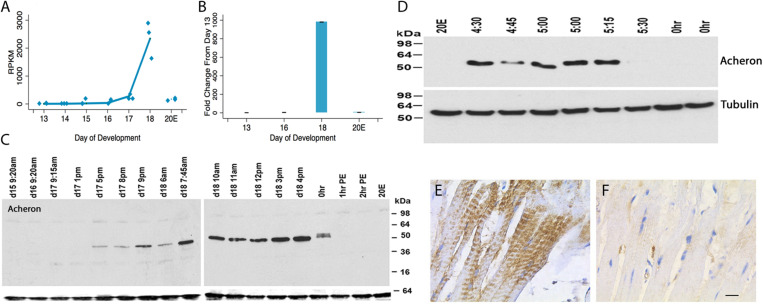
*Acheron* expression during ISM development. **(A)** RNA-seq analysis of *Acheron* mRNA in the ISMs from day 13 until day 18, the day of adult eclosion. “20E” represents animals that were injected on day 17 to delay the time of cell death and then assayed on day 18. *Acheron* expression changed during development and was induced on day 18 (*p* = 0.002) but blocked by pre-treatment with 20E (*p* = 0.025, *n* = 3 independent replicates for each stage of development). **(B)** qPCR quantification of *Acheron* mRNA late in development and following 20E treatment. Only the day 18 sample was significantly different from day 13 (*p* = 0.02). (It should be noted that these data agree well with our independent validations of this RNA-seq dataset using qPCR; Tsuji et al., 2020). **(C)** (Left) Western blot of Acheron expression at carefully timed stages between day 15 of pupal/adult development and 2 h post-eclosion (PE). Acheron expression was prevented by treatment with 20E. The blots were also probed for tubulin (bottom), which served as a loading control. **(D)** Detailed examination of Acheron expression on late day 18 starting at 4:30 p.m. Animals in the colony eclose at about 5:30 p.m. **(E,F)** Immunohistochemical staining of Acheron before **(E)** and after **(F)** adult eclosion.

*Acheron* was cloned as a novel cell death-associated gene from the ISMs (Msex001882) (Valavanis et al., 2007). Both RNA-seq and qRT-PCR demonstrated that *Acheron* mRNA is induced ∼1000-fold on day 18 of pupal–adult development when the ISMs are committed to die (Figures 1A,B). Since its expression is induced when the circulating levels of 20E decline at the end of metamorphosis (Valavanis et al., 2007), it was not surprising that treating day 17 animals with 20E blocked the anticipated induction of *Acheron* mRNA on day 18. Given the dramatic induction of *Acheron* expression, we hypothesized that it is part of the cellular killing mechanism, an idea that we rejected once we examined Acheron expression at the protein level.

Expression of Acheron protein initially tracked the abundance of its transcript. It was first detected late on day 17 (5 p.m.) when the muscles became committed to die (Figure 1C) (Schwartz and Truman, 1983). Expression continued throughout the day, but then abruptly disappeared coincident with adult eclosion. This loss was unexpected since *Acheron* mRNA persisted and no other proteins are known to be lost at the time of eclosion. To obtain a more detailed time course, we sampled muscles from animals just before or after adult eclosion (Figure 1D). Acheron was present in the ISMs until almost 5:30 p.m., but then disappeared from the cells essentially coincident with the initiation of the eclosion behavior. This was a simultaneous and global loss of Acheron throughout the tissue since we analyzed entire muscle groups rather than individual fibers. The loss of Acheron could also be visualized via immunohistochemistry using ISMs collected before and after eclosion (Figures 1E,F). In agreement with the Western blots, Acheron was essentially undetectable following eclosion.

### Acheron Phosphorylation

**FIGURE 2:**
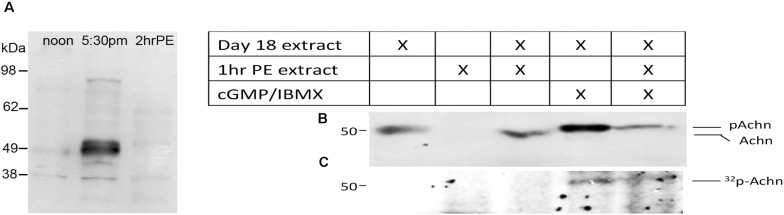
Phosphorylation and degradation of Acheron. **(A)** ISM Western blot with an anti-phospho-serine/threonine antibody detects a protein doublet the same size as Acheron at the time of eclosion. Comparable bands were not detected before or after this time point. **(B)** *In vitro* phosphorylation and degradation of Acheron. Endogenous Acheron is present in day 18 ISM extracts but not in extracts from post-eclosion (PE) muscles. Mixing the two extracts failed to induce Acheron degradation. The addition of cGMP and IBMX resulted in a higher molecular weight Acheron protein that was then susceptible to degradation when subsequently exposed to PE ISM extracts. **(C)** This experiment was repeated in the presence of ^32^P and then analyzed by film autoradiography, which demonstrated that Acheron is phosphorylated prior to degradation. These experiments were performed four times.

A careful examination of the developmental Western blot in Figure 1C reveals that the Acheron appeared as a higher molecular weight doublet just at the time of eclosion. One possible explanation for this alteration is that the protein underwent a post-translational modification prior to degradation. Further support for this hypothesis came from using an anti-phospho-serine/threonine antibody to probe a Western blot of ISM extracts (Figure 2A), which revealed a doublet of staining at ∼50 kDa, the size of Acheron in the 5:30 p.m. sample. A comparable phosphoprotein was not detected at other stages.

Since ISM death is dependent on the production of cGMP (Schwartz and Truman, 1984), we hypothesized that Acheron becomes phosphorylated by a cGMP-dependent protein kinase [protein kinase G (PKG)] at the time of eclosion and that this modification is required for its degradation. Consequently, we next employed a broken cell preparation to determine if Acheron becomes phosphorylated prior to degradation. As anticipated, pre-eclosion ISM extracts contained endogenous Acheron whereas it was absent from the PE extracts (Figure 2B). These data indicate that Acheron becomes susceptible to a proteolytic pathway following eclosion. Combining pre- and PE ISM extracts in a 1:2 ratio failed to induce Acheron degradation in the pre-eclosion sample (Figure 2B), suggesting that Acheron needs to be modified in order to render it susceptible to degradation. In separate assays day 18 extracts were supplemented with exogenous dibutyryl-cGMP to stimulate PKG, and IBMX to inhibit the phosphodiesterases that can degrade cGMP, and observed that this led to an increase in the apparent molecular weight of Acheron, consistent with phosphorylation (Figure 2B). We repeated this experiment in the presence of ^32^P-ATP followed by film autoradiography and Western blotting and observed a radiolabeled band that coincided with Acheron (Figure 2C). The addition of PE extracts to the cGMP/IBMX-treated ISM samples resulted in a dramatic reduction in the abundance of both Acheron (Figure 2B) and phospho-Acheron (Figure 2C), supporting the hypothesis that phosphorylation is essential for Acheron degradation.

**FIGURE 3:**
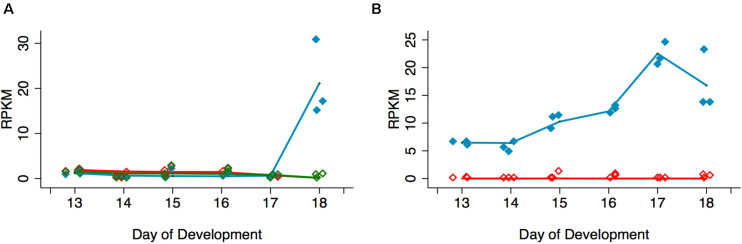
Quantification of signal transduction genes during ISM development. **(A)** RNA-seq analysis of *Eclosion Hormone Receptor* (EHR) and *Ecdysis Triggering Hormone Receptor*s (ETHR) *A* and *B* across ISM development. RNA-seq analysis of *EHR* (blue), *ETHR-A* (red), and *ETHR-B* (green). *EHR* was significantly induced on day 18 (*p* < 0.001, *n* = 3 independent replicates for each stage of development). **(B)** Expression of *cGMP-dependent protein kinase* (PKG) (blue) and *cAMP-dependent protein kinase* (red) across the ISMs across development. *PKG* was induced during development (*p* = 0.002, *n* = 3 independent replicates for each stage of development).

In support of the hypothesis that Acheron is degraded in a cGMP-dependent manner, we examined the expression of the EH Receptor (EHR), a guanylate cyclase related to the atrial natriuretic membrane receptor (Schwartz and Truman, 1984; Chang et al., 2009). An analysis of our RNA-seq data demonstrated that expression of the *EHR* (Msex003287) increased 34.6-fold between days 17 and 18 (Figure 3A). This finding is consistent with earlier studies indicating that the ability of the ISMs to respond to EH does not occur until day 18 (Schwartz and Truman, 1984). Since a distinct hormone, Ecdysis Triggering Hormone (ETH), is responsible for some ecdysis-associated physiology, such as the release of EH (Žitňan et al., 2002), we wanted to verify that ISM death is actually triggered by EH rather than ETH. RNA-seq analysis demonstrated that the *ETH Receptors A* and *B* (Msex014284 and Msex014285) were not induced at any stage (Figure 3A).

Since experimental data suggest that Acheron is phosphorylated at eclosion in a cGMP-dependent manner, we also wanted to evaluate the expression of *PKG* (Msex002121). In contrast to cAMP-dependent protein kinase (Msex002485), which was essentially undetectable at any stage of development examined, *PKG* expression increased prior to adult eclosion (Figure 3B). These data suggest that EH, acting through the EHR, could trigger Acheron phosphorylation in a cGMP-dependent manner.

### BBH1 Is an Acheron Binding Protein

**FIGURE 4:**
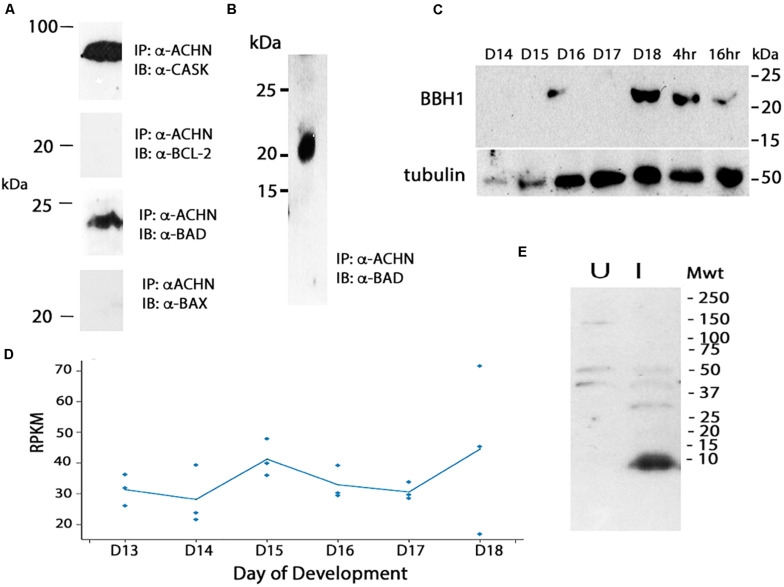
Cloning and analysis of BBH1. **(A)** Co-immunoprecipitation (co-IP) assays were used to identify binding partners in mouse C_2_C_12_ myoblasts. Acheron binds to CASK, a previously identified partner, but not the pro-survival protein Bcl-2. It does bind to the BH3-only protein BAD, but not the related protein BAX. **(B)** Co-IP assays with day 18 ISM extracts demonstrate that Acheron binds to a protein that cross-reacts with an anti-mouse BAD antibody. **(C)** Expression of BBH1 protein across ISM development. Tubulin was used as a loading control. **(D)** RNA-seq quantification of *BBH1* expression during development. There was no significant change (*p* = 0.28, *n* = 3 independent replicates for each stage of development). **(E)** The anti-BAD antibody recognizes a bacterially expressed portion of *Manduca* BBH1 protein in the induced (“I”) sample but not the uninduced sample (“U”).

We hypothesized that the degradation of Acheron liberates a pro-death protein that then triggers ISM death. In considering potential candidates, we turned to the control of apoptosis, where death often employs pro-apoptotic Bcl-2 Homology 3 (BH3)-only family proteins (reviewed in Doerflinger et al., 2015). As a first step in this analysis, we utilized mouse C_2_C_12_ myoblasts since Acheron is abundantly expressed in these well characterized cells and has been shown to regulate myogenesis (Wang et al., 2009). Co-immunoprecipitation assays (co-IP) were performed with an anti-Acheron antibody followed by Western blotting for candidate partners. As a positive control, we demonstrated that Acheron bound to the signaling protein CASK, a known binding partner (Weng et al., 2009) (Figure 4A). There was no detectable interaction between Acheron and the anti-apoptotic protein Bcl-2. However the pro-apoptotic protein BAD, which can drive cell death in a variety of cell types (Yang et al., 1995), is an Acheron binding partner (Figure 4A). [We separately verified this interaction by performing co-IP assays using human HCC1935 breast cancer cells (data not shown).] The interaction between Acheron and BAD was specific since the related BH3-only protein BAX failed to bind. Thus, we can directly tie the function of Acheron to well-established cell death programs via binding to BAD.

We next performed co-IP experiments in the ISMs with Acheron and a commercial anti-BAD antibody, and demonstrated a physical interaction between Acheron and a 21 kDa BAD-like protein (Figure 4B). Western blotting demonstrated that this BAD ortholog was induced on day 18 when the ISMs were committed to die, and then persisted throughout death (Figure 4C). The possible identity of this protein was unknown since insects are reported to lack BH3-only proteins (e.g., Nicolson et al., 2015). Consequently, we conceptually translated our RNA-seq database and identified all proteins that: (1) are ∼21 kDa; (2) are expressed more than 10 RPKM at some stage of development; and (3) have motifs with homology to BH3 domains. We identified a single candidate that was computationally identified as BNIP3 (Bcl-2 adenovirus E1B 19 kDa-interacting protein 3) (Msex012014). BNIP3 is a BH3-only protein that localizes to mitochondria and can induce cytochrome *c* release and drive both apoptosis and autophagy in a variety of mammalian tissues (Vande Velde et al., 2000; Vasagiri and Kutala, 2014; Gao et al., 2020). The *Manduca* protein is 21.3 kDa and shares several domains with sequence similarity with both mouse BAD and BNIP3 (Supplementary Figure 1), including a dimerization/membrane insertion domain. We have named this BH3-only protein *Manduca* BBH1 (BAD/BNIP3 homology-1). *BBH1* mRNA appears to be constitutively expressed in the ISMs, although there was variability in the expression on day 18 (Figure 4D).

To verify that the anti-mouse BAD antibody directly recognizes BBH1, we subcloned a portion of the BBH1 cDNA into a bacterial expression vector and observed that it was labeled on Western blot (Figure 4E). (We have tried several methods to express the full length protein for physiological studies but have been unsuccessful because it appears to be toxic to *E. coli*).

To insure that the anti-mouse BAD antibody was not recognizing BNIP3 in mouse C_2_C_12_ myoblasts, we generated Western blots with control and hypoxic (CoCl_2_-treated) C_2_C_12_ myoblasts and probed them with antibodies against BAD and BNIP3. BAD expression was repressed by hypoxia while BNIP3 was dramatically induced as anticipated (Zhu et al., 2019), suggesting that our anti-BAD antibody does not recognize BNIP3 (Supplementary Figure 2).

### Genetic Interventions to Examine the Roles of Acheron and BBH1 in Ecdysial Muscle Degeneration

**FIGURE 5:**
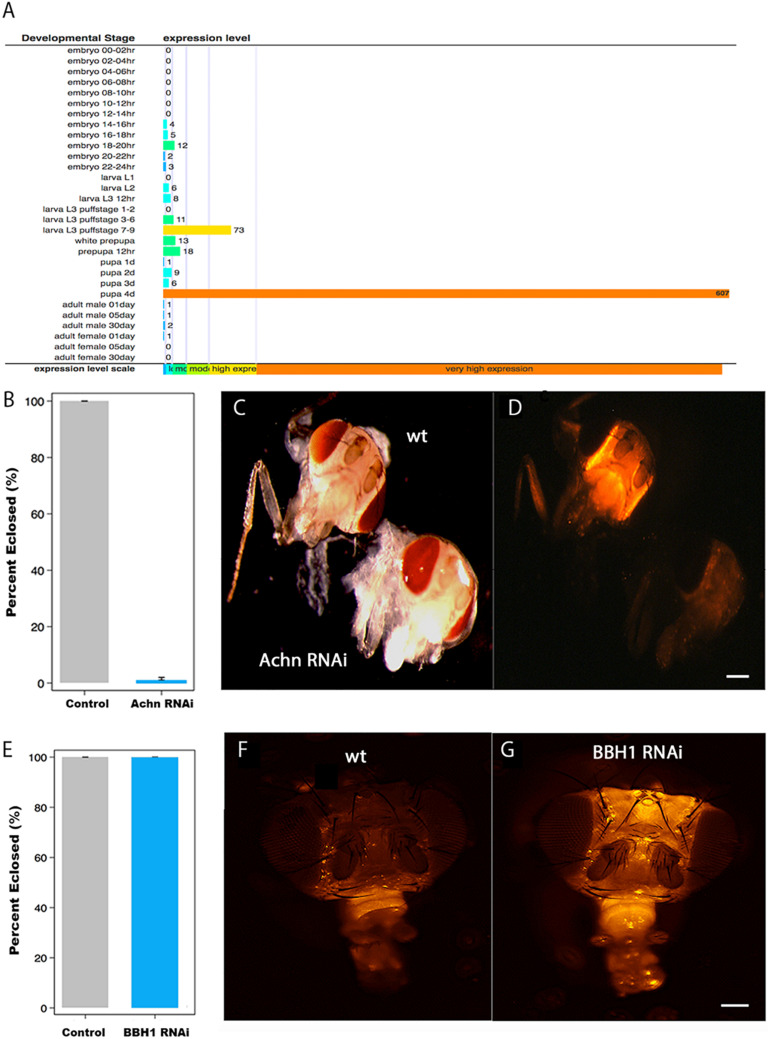
The role of Acheron and BBH1 on ecdysial muscle death in *Drosophila*. **(A)** Data from Flybase modENCODE Tissue Expression Data based on RNA-seq analysis (CG17386). *Acheron* is transiently induced ∼600-fold just before adult eclosion. **(B)** Percentage of *Drosophila* that eclosed successfully in control and *Acheron* RNAi expressing animals. *Acheron* RNAi blocked eclosion (*p* < 0.0001, mean ± SE, *n* = 28 and 202, respectively). **(C)** Pre-eclosion pharate adult transgenic flies expressing red fluorescent protein (RFP) in the skeletal muscles of control (top) and Acheron (CG17386) RNAi flies. **(D)** Fluorescent imaging of the same animals showing that Acheron RNAi knockdown led to the precocious death of the ptilinal muscles. **(E)** Percentage of flies that eclosed successfully in control and *BBH1* RNAi animals (*p* = 1.0, mean ± SE, n = 28 and 154, respectively). **(F)** Fluorescent image of a control fly head 36 h post-eclosion showing the normal loss of the ptilinal muscles. **(G)** Fluorescent image of a BBH1 RNAi fly head 36 h post-eclosion showing the retention of the ptilinal muscles. Note that the muscles in the proboscis (bottom front of the heads) persist in the adult. wt, wild type. Scale bars = 100 μm.

The data presented above suggest that the loss of Acheron, and the presumptive liberation of BBH1, act to trigger ISM cell death. Ideally, to test this hypothesis directly, we would employ genetic interventions to disrupt *Acheron* and *BBH1* expression to determine if this alters the timing of cell death. *Manduca* is not well suited for genetic manipulations (long generation time, few genetic markers, 28 chromosomes, etc.), and unfortunately, RNAi does not work in this species (Garbutt et al., 2013). Consequently, we turned to the fruit fly *Drosophila melanogaster*, where powerful tools are available for the targeted regulation of gene expression *in vivo* (Perkins et al., 2015). As a first step, we needed to verify that *Drosophila* has an *Acheron* gene and that it is induced at the time of adult eclosion. The fly *Acheron* gene (CG17386) [also known as Achilles (Li et al., 2017)] is dramatically induced (∼600-fold) just prior to adult eclosion and is then no longer detectable (Figure 5A).

Unlike Lepidoptera, which rely on abdominal muscles to generate the eclosion behavior, Diptera employ the ptilinal muscles in the head (Laing, 1935). Since there are no reported Gal4-UAS promoters that target gene expression specifically to the ptlinal muscles, we used the general muscle driver DMEF-Gal4 to express Acheron RNAi in all of the muscles throughout development. To better visualize the muscles, we co-expressed RFP using the UAS-dsRED reporter. Both the control (wild type; wt) and Acheron RNAi flies developed normally and formed healthy pharate adult animals, suggesting that the chronic repression of Acheron expression in all muscles had no discernable impact on development. However, the loss of Acheron prevented the animals from being able to generate the eclosion behavior and escape the puparium (Figure 5B). Control animals displayed strong dsRED fluorescence in their heads (Figures 5C,D, upper animal), while the Acheron RNAi flies did not, thus demonstrating the precocious loss of the ptilinal muscles.

To extend these analyses, and examine the role of BBH1 in the regulation of ptilinal muscle PCD, we created transgenic flies that co-expressed RFP and *Drosophila BBH1*-RNAi (CG5059) and evaluated both the performance of the eclosion behavior and muscle loss. Unlike Acheron knock-down which blocked eclosion, *BBH1* RNAi flies emerged at wild-type levels, suggesting that the ptilinal muscles were functional (Figure 5E). However, while the ptilinal muscles of wild-type flies underwent the normal process of PCD by 36 h PE as anticipated (Figure 5F), flies expressing *BBH1* RNAi still retained their muscles (Figure 5G). Taken together, these data suggest that BBH1 acts downstream of Acheron and directly mediates the death of ecdysial muscles.

### Cytochrome c Release and Degradation

**FIGURE 6:**
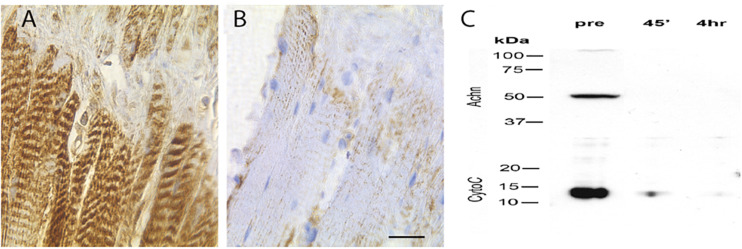
Expression of cytochrome *c* in the ISMs. Cytochrome *c* immunohistochemical staining of ISMs demonstrates abundant punctate staining in the ISMs prior to adult eclosion **(A)**, which was lost throughout the tissue following eclosion **(B)**. The nuclei are stained blue. Scale bar = ∼280 μm. **(C)** Western blot analysis of Acheron and cytochrome *c* in the ISMs before and after adult eclosion.

BH3-only proteins act primarily via their ability to insert into the mitochondrial membrane and induce the release of cytochrome *c* (Quinsay et al., 2010; Vasagiri and Kutala, 2014). To determine if this could be part of the molecular mechanism that mediates ISM death, we stained ISM tissue sections with an anti-cytochrome *c* antiserum. We observed intense punctate staining along the length of the fiber on day 18 that labeled the repeated clusters of mitochondria (Figure 6A). In contrast, cytochrome *c* staining was lost following eclosion (Figure 6B). (This differential staining was not an artifact of processing since the sections from both the pre- and PE ISM muscles were present on the same glass slides).

During the initiation of apoptosis, cytochrome *c* transitions from puncate mitochondrial staining to diffuse cytoplasmic localization as the protein is released (Goldstein et al., 2005). Cytoplasmic cytochrome *c* persists and facilitates the formation of the apoptosome and the subsequent activation of pro-caspase 9 (Liu et al., 1996). However, instead of displaying cytoplasmic staining when the ISMs initiate death, cytochrome *c* disappears, suggesting that it had been degraded. To independently confirm this observation, and insure that this is not an artifact from regional sampling, we employed Western blotting to biochemically evaluate the relative abundance cytochrome *c* in the whole tissue. As anticipated, pre-eclosion ISMs contained both Acheron and cytochrome *c*, but neither protein was present in the PE sample (Figure 6C), consistent with degradation.

## Discussion

**FIGURE 7:**
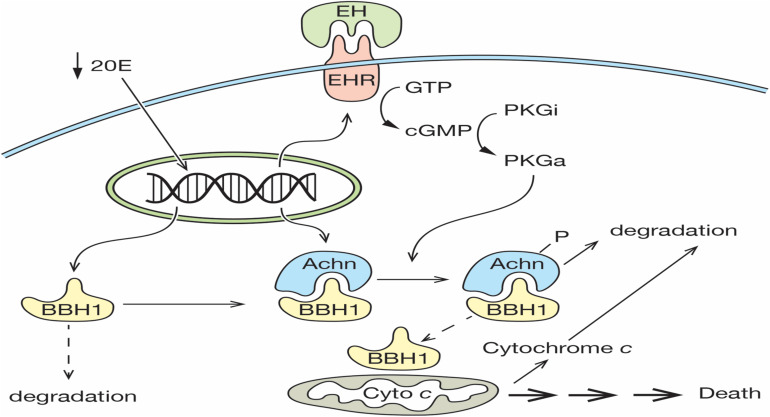
Model for ISM cell death. On day 17 of pupal–adult development, the circulating levels of 20E decline below a threshold that triggers the expression of both the Eclosion Hormone Receptor (EHR) and Acheron. Acheron binds to and stabilizes the pro-death protein BBH1, which then accumulates. A further decline of 20E on day 18 triggers the release of eclosion hormone, which binds to the EHR and drives the production of cGMP, the conversion of inactive protein kinase G (PKGi) into PKG active (PKGa), and Acheron phosphorylation, which leads to its degradation. This liberates BBH1, which then induces the release and degradation of cytochrome *c* from mitochondria and the subsequent non-apoptotic death of the muscles. (Solid lines denote events that have been demonstrated experimentally, while dashed lines have not been formally tested).

The death of the ISMs represents one of the classic examples of PCD, yet the molecular mechanism that mediates this cell loss is largely unknown. We propose that the expression and subsequent degradation of Acheron is the essential upstream regulatory event that controls ISM loss. In our model, a decline in the circulating titer of 20E late on day 17 primes the cell for death by inducing the expression of both Acheron and the EHR (Figure 7). Acheron then binds to and stabilizes the pro-apoptotic protein BBH1, a constitutively expressed protein that has a short half-life due to a PEST sequence that targets its degradation (Cizeau et al., 2000). Since BH3-only proteins can directly induce cell death in a range of cell types (Kanzawa et al., 2005; Maiuri et al., 2007; Shi et al., 2014; Pedanou et al., 2016; Gao et al., 2020), the stabilization and accumulation of BBH1 has the effect of making the muscles dependent on the continued presence of Acheron for survival, reminiscent of the “addiction modules” of bacteria (Engelberg-Kulka and Glaser, 1999). A further decline of 20E late on day 18 triggers the release of EH, which acts on the nervous system to generate the eclosion motor program that helps drive the animal out of the pupal cuticle (Truman and Sokolove, 1972; Truman et al., 1983). It also acts directly on the ISMs via the EHR to induce the synthesis of the second messenger cGMP, which has been shown to be an essential step in triggering ISM death (Schwartz and Truman, 1984). *In vitro* assays have demonstrated that Acheron phosphorylation can be induced in broken cell preparations with exogenous cGMP (Figure 2). Much like the proteasomal degradation of phosphorylated beta-catenin (Stamos and Weis, 2013), Acheron phosphorylation appears to be essential to render it susceptible to breakdown. The fact that PE extracts cannot degrade Acheron in the absence of phosphorylation supports this conclusion.

The rapid degradation of Acheron presumably liberates its binding partners within the cytoplasm, including BBH1. One of the best characterized roles for BH3-only proteins is to oligomerize and form pores in mitochondria, which in turn facilitates the rapid release of cytochrome *c* (Quinsay et al., 2010; Vasagiri and Kutala, 2014). BBH1 has a domain in the C-terminus (amino acids 157–178; Supplementary Figure 1) with high sequence and structural identity with a domain in BNIP-3 that functions as a dimerization domain that inserts into mitochondrial outer membranes (Hendgen-Cotta et al., 2017). Since BNIP-3 can directly induce the release of cytochrome *c*, we would predict that BBH1 could serve a similar function in the ISMs. We tried to test this hypothesis directly using purified BBH1 and isolated ISM mitochondria, but we have been unable to express the protein in bacteria due to its apparent toxicity (unpublished). Consequently, a direct test of this hypothesis awaits further examination. It is interesting to note that in cardiac muscle, a tissue that expresses high levels of Acheron, the application of exogenous cGMP can induce the release of cytochrome *c* (Valavanis et al., 2007; Seya et al., 2013). Whether or not the pathway involved utilizes the same machinery as we have described for the ISMs has not been elucidated.

One of the primary consequences of cytochrome *c* release is the formation of the apoptosome and the sequential activations of pro-caspase 9 and downstream executioner caspases like caspase-3 (Zou et al., 1997; Hu et al., 1998). This results in a range of phenotypic changes associated with apoptosis. The ISMs display none of these features and instead die by a process that has been termed type II degeneration or autophagic cell death (Beaulaton and Lockshin, 1977; Schwartz et al., 1993; Schwartz, 2019). More recently, this program of cellular suicide has been referred to as autophagic dependent cell death (ADCD) (Galluzzi et al., 2018). This form of cell death is seen with some neurons, muscles, and certain cancers (Gama et al., 2014; Marino et al., 2014). It has been reported that the rapid degradation of cytochrome *c* in cells that express low Apf-1 levels, like muscles, precludes apoptosome formation, thus rendering them apoptosis-resistant cells (Gama et al., 2014). The inability to initiate apoptosis may allow other slower cell death programs that were triggered concurrently to be revealed, including ADCD. The rapid degradation of cytochrome *c* observed in the ISMs may serve to ensure that apoptosis is not triggered. This may be advantageous given that the ISMs are composed of giant cells (each one of which is ∼5 mm long and up to 1 mm in diameter) and the hemolymph of *Manduca* does not possess enough phagocytic cells to engulf and degrade them (Jones and Schwartz, 2001). Consequently, the use of autophagy and the ubiquitin-proteasome pathway may represent a more efficient mechanism for recycling muscle macromolecules for use by the adult (Haas et al., 1995).

Acheron is a phylogenetically conserved protein that plays a number of critical roles in both development and pathogenesis (Stavraka and Blagden, 2015). It is required for differentiation and cell survival in several lineages in vertebrates, including muscle, neurons, osteoblasts (Wang et al., 2009; Guo et al., 2017), and multi-ciliated cells (Manojlovic et al., 2017). It also confers a growth advantage for some tumors, such as triple-negative basal-like breast cancers, where it helps drive angiogenesis and invasiveness (Shao et al., 2012). Acheron also protects rheumatoid arthritis fibroblastic synoviocytes from apoptosis induced by the death ligand TRAIL (Audo et al., 2015). Taken together, these various lines of evidence suggest that Acheron functions as a novel survival protein that can protect cells from cell death. Given that Acheron is predominantly expressed in terminally differentiated cells, it may also function to protect cells in these lineages from pro-death insults.

## Data Availability Statement

All the sequencing libraries are accessible from Gene Expression Omnibus (GEO) (accession number GSE80830).

## Ethics Statement

No vertebrate animals or human subjects data were used for this study.

## Author Contributions

AS, RS, and CBr generated the majority of the *Manduca* and mouse myoblast data. DS made the anti-Acheron antibody. JJ, AH, and MM performed the *Drosophila* experiments. WS and PV contributed to the phosphorylation experiments. CBi performed the statistical analyses. LS oversaw the study and wrote the manuscript. All authors contributed to the article and approved the submitted version.

## Conflict of Interest

The authors declare that the research was conducted in the absence of any commercial or financial relationships that could be construed as a potential conflict of interest.

## Footnotes

1 http://agripestbase.org/manduca/

## Supplementary Material

FIGURE S1 *Manduca* BBH1 sequence analysis. The sequence of *Manduca* BBH1 (middle) is compared to mouse BAD (top) and mouse BNIP3 (bottom). BBH1 shares 16.7% identity/70.0% similar in a 90 aa overlap (36–123:44–133) with BAD and 23.4% identity/62.0% in a 192 aa overlap (10–181:7–179) with BNIP3. Identical sequences are denoted by yellow, while conserved substitutions are highlighted in blue. Amino acid residues that are not conserved between BAD and BNIP3, but are conserved with BBH1, are highlighted in green. The putative PEST sequence of BBH1 is underlined in red while the presumptive transmembrane/dimerization domain is in bold. The amino acid positions are shown on the right.

FIGURE S2 Anti-BAD antibody recognizes BAD but not BNIP3. **(A)** Expression of BAD and **(B)** BNIP3 in C_2_C_12_ myotubes. Cells were cultured in normal growth medium (left) or exposed to 100 μM CoCl_2_ for 24 h to induce hypoxia and then Western blots were probed with the appropriate antisera.
