# Supplementary figures and images for: Acheron/Larp6 Is a Survival Protein That Protects Skeletal Muscle From Programmed Cell Death During Development
Source: Front Cell Dev Biol. 2020 Jul 29;8:622. doi: 10.3389/fcell.2020.00622 (PMC7405549; doi:10.3389/fcell.2020.00622)

**
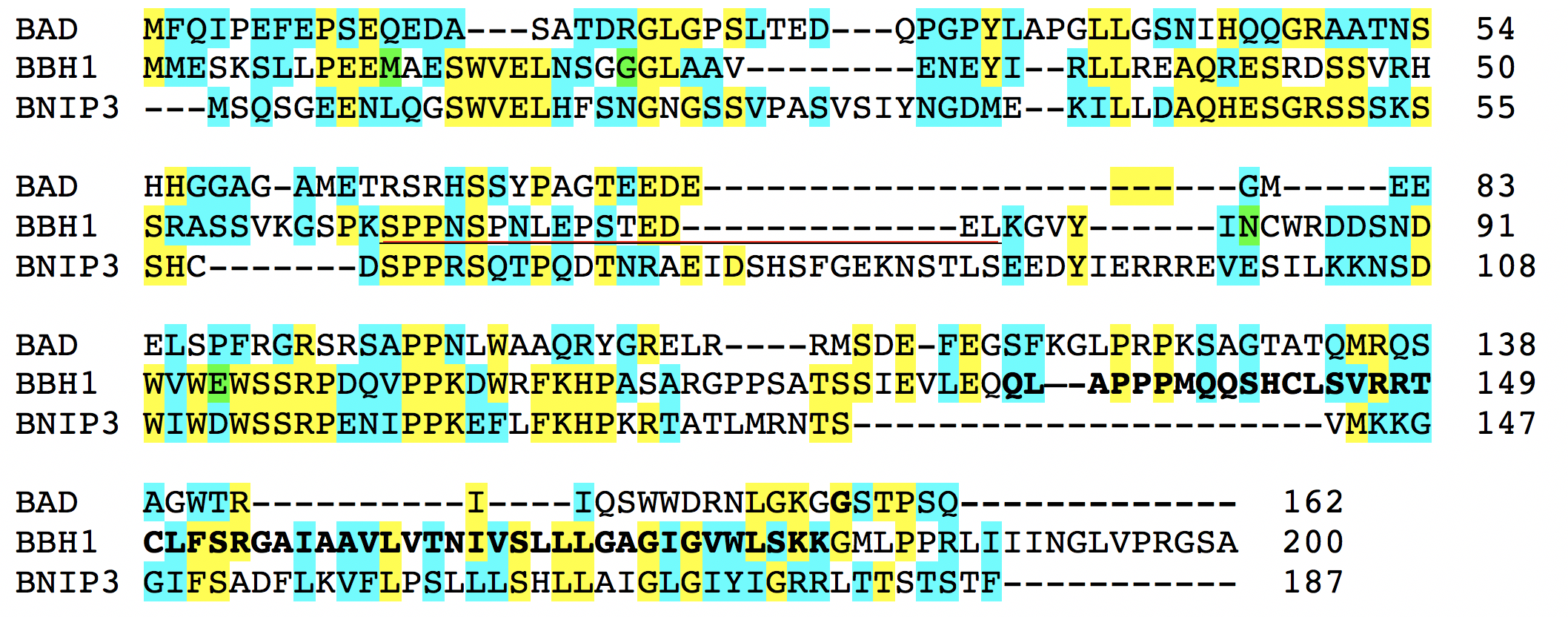
**

Supplement: FIGURE S1 — Manduca BBH1 sequence analysis. The sequence of Manduca BBH1 (middle) is compared to mouse BAD (top) and mouse BNIP3 (bottom). BBH1 shares 16.7% identity/70.0% similar in a 90 aa overlap (36–123:44–133) with BAD and 23.4% identity/62.0% in a 192 aa overlap (10–181:7–179) with BNIP3. Identical sequences are denoted by yellow, while conserved substitutions are highlighted in blue. Amino acid residues that are not conserved between BAD and BNIP3, but are conserved with BBH1, are highlighted in green. The putative PEST sequence of BBH1 is underlined in red while the presumptive transmembrane/dimerization domain is in bold. The amino acid positions are shown on the right. [file Table_1.DOCX]

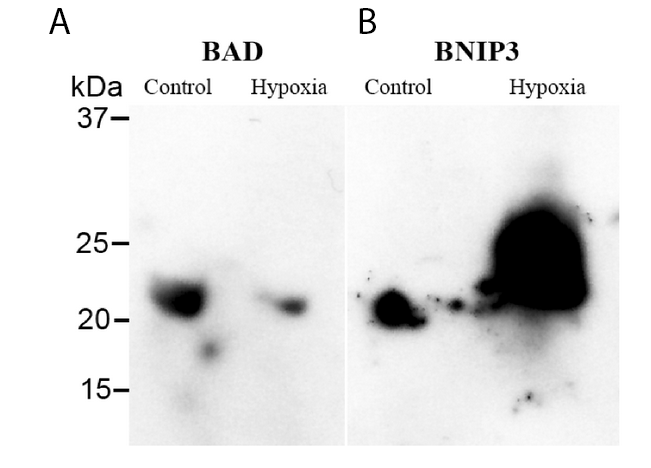

Supplement: FIGURE S2 — Anti-BAD antibody recognizes BAD but not BNIP3. (A) Expression of BAD and (B) BNIP3 in C2C12 myotubes. Cells were cultured in normal growth medium (left) or exposed to 100 μM CoCl2 for 24 h to induce hypoxia and then Western blots were probed with the appropriate antisera. [file Image_1.TIF]
